# *Mucilaginibacter aquariorum* sp. nov., Isolated from Fresh Water

**DOI:** 10.4014/jmb.2208.08021

**Published:** 2022-10-31

**Authors:** Ve Van Le, So-Ra Ko, Mingyeong Kang, Hee-Mock Oh, Chi-Yong Ahn

**Affiliations:** 1 Cell Factory Research Center, Korea Research Institute of Bioscience and Biotechnology (KRIBB), Daejeon 34141, Republic of Korea; 2 Department of Environmental Biotechnology, KRIBB School of Biotechnology, University of Science and Technology (UST), Daejeon 34113, Republic of Korea

**Keywords:** *Mucilaginibacter aquariorum*, fresh water, novel species

## Abstract

A Gram-stain-negative, rod-shaped bacterial strain, JC4^T^, was isolated from a freshwater sample and determined the taxonomic position. Initial identification based on 16S rRNA gene sequences revealed that strain JC4^T^ is affiliated to the genus *Mucilaginibacter* with a sequence similarity of 97.97% to *Mucilaginibacter rigui* WPCB133^T^. The average nucleotide identity and digital DNA–DNA hybridization values between strain JC4^T^ and *Mucilaginibacter* species were estimated below 80.92% and 23.9%, respectively. Strain JC4^T^ contained summed feature 3 (C_16:1_ *ω7c* and/or C_16:1_ *ω6c*) and iso-C_15:0_ as predominant cellular fatty acids. The dominant polar lipids were identified as phosphatidylethanolamine, one unidentified aminophospholipid, one unidentified phospholipid, and two unidentified lipids. The respiratory quinone was MK-7. The genomic DNA G+C content of strain JC4^T^ was determined to be 42.44%. The above polyphasic evidences support that strain JC4^T^ represents a novel species of the genus *Mucilaginibacter*, for which the name *Mucilaginibacter aquariorum* sp. nov. is proposed. The type strain is JC4^T^ (= KCTC 92230^T^ = LMG 32715^T^).

## Introduction

The genus *Mucilaginibacter* was described for the first time with the type species *Mucilaginibacter paludis* by Pankratov *et al*. [1]. Phylogenetically, this genus is affiliated with the family *Sphingobacteriaceae* in the class *Sphingobacteriia* [1]. Currently, at least 74 validly published species have been isolated from diverse ecological niches, such as phycosphere of microalgae *Haematococcus* [2], fresh water [3], marine sand [4], sediment [5], and soil [6]. Members of the genus *Mucilaginibacter* were identified as Gram-negative, non-motile, and rod-shaped [1]. They are known to produce extracellular polymeric substances [7], bioremediate heavy metal contamination [8], improve growth and salt tolerance in plants [9], and degrade cellulose [10].

Freshwater pollution seriously threatens aquatic ecosystems and human health [11]. Microbial communities may shift their structure, function, and interaction network in response to environmental contamination [12]. As a part of the study on the potential use of bacteria communities as indicators of aquatic ecosystem state, strain JC4^T^ was isolated from a freshwater sample of Jochon Stream. This study aimed to determine the taxonomic status of strain JC4^T^.

## Materials and Methods

### Bacterial Isolation and Cultivation

Strain JC4^T^ was isolated from a freshwater sample that was collected at Jochon Stream (35o 52′ 57′′ N, 127o 03′47′′ E), Jeonju, South Korea, in October 2021. The strain was purified, routinely cultivated, and preserved as previously described [13]. Based on 16S rRNA gene sequence similarity, *Mucilaginibacter rigui* KCTC 12534^T^ (= WPCB133^T^) [14], *Mucilaginibacter lutimaris* KCTC 23461^T^ (= BR-3^T^) [5], and *Mucilaginibacter terrigena* KCTC 62294^T^ (= 17JY9-4^T^) [15] were selected as reference strains for comparative polyphasic characterization. All related type strains were purchased from the Korean Collection for Type Cultures. Since strain JC4^T^ and reference strains grew well on R2A medium, their phenotypic and chemotaxonomic features were investigated using routine cultivation on this medium.

### Phylogenetic Analysis

Genomic DNA was extracted from three-day-old cultures according to Ko *et al*. [16]. Amplification and sequencing of the 16S rRNA gene were performed using universal primer set 27F (5'-AGAGTTTGATCATGGCTCAG-3') and 1492R (5'-TACGGYTACCTTGTTACGACTT-3') [17]. The nearly full-length 16S rRNA gene sequence of strain JC4^T^ (1,402 bp) was compared with those of valid species in the EzTaxon server. Phylogenetic trees were constructed using neighbor-joining (NJ) [18], maximum-likelihood (ML) [19], and minimum-evolution (ME)[20] methods in the MEGA X software [21]. The evolutionary distances were determined by Kimura 2-parameter correction [22]. The robustness of phylogenetic trees was evaluated based on 1000 bootstrap replicates.

### Genomic Analysis

The draft genome of strain JC4^T^ was analyzed as described previously [23]. Briefly, genome sequencing was performed using Illumina platform. In order to avoid bias in the analysis, low-quality reads and adapter sequences were removed using Trimmomatic (v0.36) [24]. Benchmarking Universal Single-Copy Orthologous (BUSCO, v3.0) was employed to determine the assembly quality [25]. The circular genome map was generated using the PATRIC server [26]. The genome of strain JC4^T^ was annotated by the NCBI Prokaryotic Genome Annotation Pipeline (PGAP) [27]. The protein-coding sequences were functionally assigned to Clusters of Orthologous Groups (COG) categories using the EggNOG-mapper online server [28]. The putative secondary metabolite biosynthetic gene was predicted using the online antiSMASH server [29]. The genomic DNA G+C content was obtained directly from the genomic sequence.

ANI Calculator [30] and Genome-to-Genome Distance Calculator formula-2 [31] were applied to calculate the average nucleotide identity (ANI) and digital DNA-DNA hybridization (dDDH) values. To further clarify the taxonomic status of JC4^T^, a phylogenomic tree was constructed using the Type (Strain) Genome Server [32]. All the genome sequences of close relatives were obtained from the GenBank database (Table 1).

### Morphological and Biochemical Features

The cell shape and size of strain JC4^T^ were examined via a transmission electron microscope (CM-20, Philips). Motility was assessed by the hanging-drop method. The Gram reaction was performed using a Gram Stain kit (BD, USA). The ability of strain JC4^T^ to grow on various media, including tryptone soy agar (TSA, Difco), nutrient agar (NA, Difco), and Luria-Bertani agar (LBA, Difco) was recorded for up to five days of incubation at 25°C. Growth was observed in R2A broth (Difco) at different pH values (4.0–12.0 at 0.5 pH unit intervals) and temperatures (4, 10, 15, 20, 25, 30, 37, and 40°C) for five days [13]. Catalase and oxidase activity were detected as described previously [2]. Metabolic activity was determined by commercial API kits (bioMérieux, France). The hydrolysis of lipid, casein, and skim milk was investigated following the methods of Smibert and Krieg [33]. Positive hydrolysis was indicated by a clear zone around colonies. Antibiotic susceptibility of strain JC4^T^ was examined by a Kirby-Bauer disc diffusion method [34] on R2A agar medium [16].

### Chemotaxonomic Analysis

The compositions of fatty acids and polar lipids of strain JC4^T^ and related type strains were obtained from bacterial cells grown on R2A medium at 25°C for three days. The cellular fatty acids were prepared and identified following the standard MIDI protocol [35]. Polar lipids were extracted and separated using two-dimensional thin-layer chromatography (TLC) on silica gel thin-layer chromatography Kieselgel 60 F_254_ plates (Merck, USA) [36-39]. Ethanolic molybdophosphoric acid (Sigma-Aldrich, USA) was sprayed on the plates to visualize the total polar lipids. In addition, amino, sugar, and phosphate groups were detected using the staining reagents ninhydrin, α-naphthol, and molybdenum blue (Sigma-Aldrich), respectively. Quinones were extracted using chloroform/methanol (2:1, v/v) and identified using high-performance liquid chromatography (LC-20A, Shimadzu, Japan) [40].

## Results and Discussion

### Phylogenetic Features

**Fig. 1:**
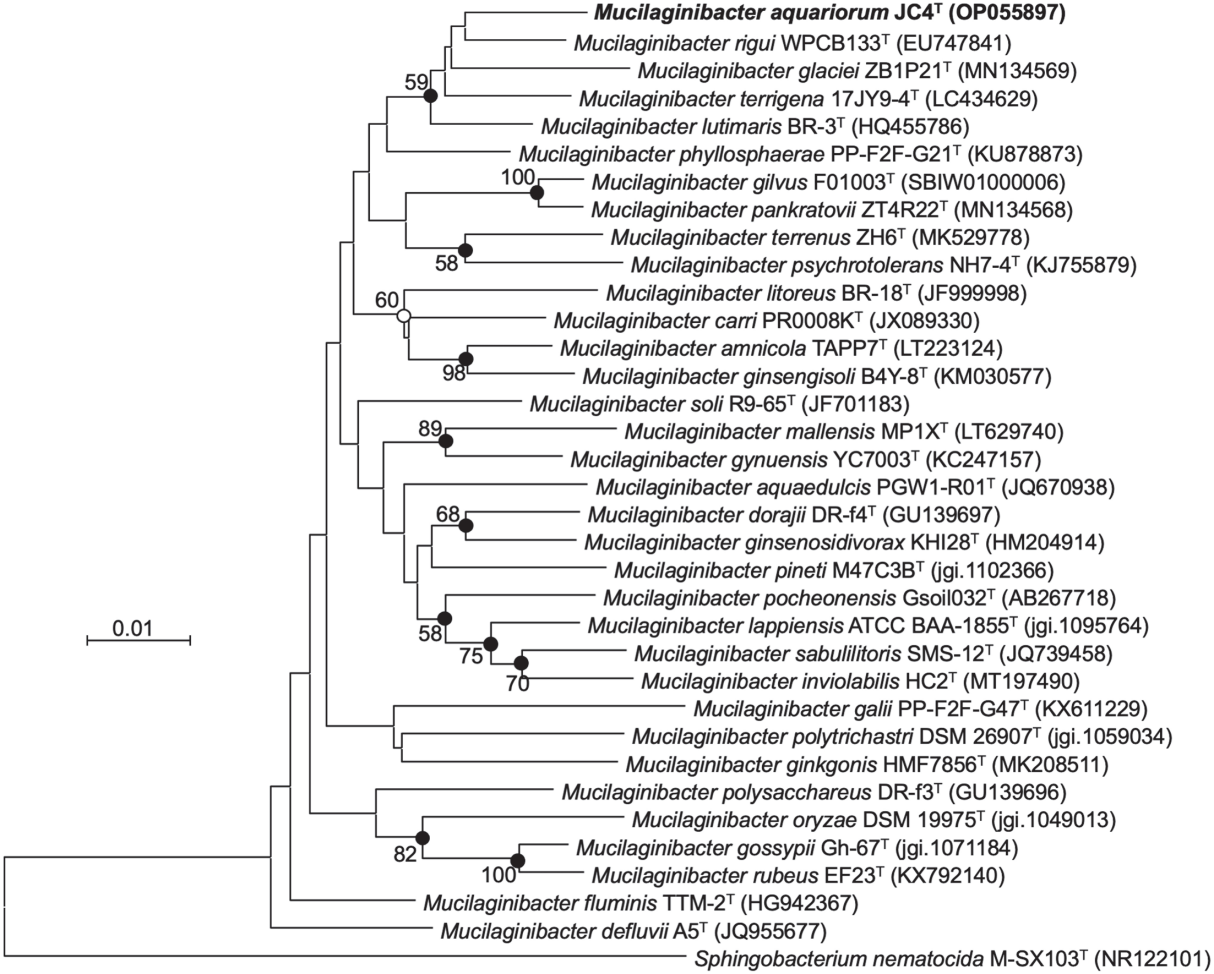
Neighbor-joining phylogenetic tree based on the 16S rRNA gene sequences depicting the phylogenetic relationships of strain JC4^T^ among the related members of the genus *Mucilaginibacter*. Bootstra*p* values (≥ 50%) based on 1,000 replications are shown at branch points. Filled circles at nodes indicate that the corresponding nodes were also recovered in the maximum-likelihood and maximum-parsimony methods whereas the nodes with open circles were recovered with NJ and ME algorithms. *Sphingobacterium nematocida* M-SX103^T^ (NR122101) was used as an outgroup. Scale bar, 0.01 nucleotide substitutions per nucleotide position.

Strain JC4^T^ shared the highest 16S rRNA sequence similarity to *M. rigui* WPCB133^T^ (97.97% similarity), *M. lutimaris* BR-3^T^ (97.83%), and *M. terrigena* 17JY9-4^T^ (97.58%). All the phylogenetic trees revealed that the strain formed a monophyletic clade with *M. rigui* WPCB133^T^ (Figs. 1, S1, and S2), supporting its affiliation to the genus *Mucilaginibacter*.

### Genomic and Phylogenomic Analyses

**Fig. 2:**
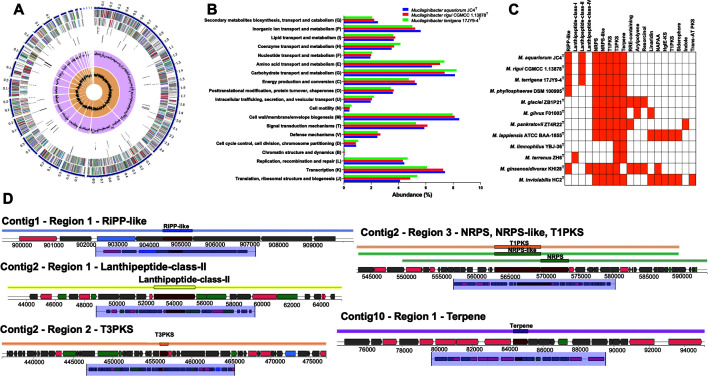
Circular view of the genome of strain JC4^T^ (**A**). From the outside to the inside: contigs; coding sequence in forward strand; coding sequence in reverse strand; RNA genes; antimicrobial resistance genes; GC content, and GC skew. Clusters of Orthologous Groups (COG) analysis of the genome of JC4^T^, *M. rigui* WPCB133^T^, and *M. terrigena* 17JY9-4^T^ (**B**). Secondary metabolite biosynthetic gene clusters predicted in the genomes of *Mucilaginibacter* species (**C**) and strain JC4^T^ (**D**). The yellow color indicates the presence of the biosynthetic gene cluster.

Strain JC4^T^ has a draft genome of 5.97 Mb, an L50 value of 2, and an N50 length of 880,985 bp (Fig. 2A and Table 1). The coverage and completeness were 126× and 98.39%, respectively. The 16S rRNA gene sequences of strain JC4^T^ obtained from Sanger sequencing were 100% identical to those from the genome, indicating the accuracy of the genome assembly. Collectively, the genome sequence of strain JC4^T^ meets the minimal requirements for the taxonomic purposes proposed [41]. The JC4^T^ genome contained a total of 5,312 protein coding sequences, 15 pseudo genes, 46 tRNAs, and 5 rRNAs. COG (Clusters of Orthologous Groups of proteins) analysis showed that JC4^T^, *M. rigui* WPCB133^T^, and *M. terrigena* 17JY9-4^T^ have the highest proportions of gene related to basic cellular processes, such as carbohydrate transport and metabolism, amino acid transport and metabolism, and post-translational modification (Fig. 2B). The genomic DNA G+C content of strain JC4^T^ was found to be 42.44%, within the range of 42.4-46.1% reported for the genus *Mucilaginibacter* [1].

Bacteria produce secondary metabolites to interact with other microorganisms and adapt to environmental changes [42]. Little is known about secondary metabolites produced by the genus *Mucilaginibacter*. T3PKS (type III polyketide synthase) was predicted in all *Mucilaginibacter* species (Fig. 2C). The genome of strain JC4^T^ harboured five putative secondary metabolite biosynthetic gene clusters involved in synthesizing RiPP (ribosomally synthesized and post-translationally modified peptide)-like product, lanthipeptide-class-II, T3PKS, terpene, and NRPS (nonribosomal polyketide synthase), NRPS-like, T1PKS (Fig. 2D). Lanthipeptide-class-II is a promising alternative to classical antibiotics and is used as probiotics, additives in cosmetics and personal-care products [43]. Plants and fungi produce terpene as part of their defense mechanism [44]. Currently, terpene synthase genes have been found to be distributed in bacteria such as *Nonomuraea terrae* [45], *Caenimonas aquaedulcis* [46], *Panacibacter microcysteis* [47], and *Nocardia noduli* [48], which may be useful for discovering new natural products [49]. The presence of NRPS in the genome of strain JC4^T^ suggested that this strain can produce antimicrobial, antiviral, anti-cancer, and anti-inflammatory compounds [50]. Several bacteria can produce cold-shock proteins in response to sudden temperature decreases [51]. The genome of strain JC4^T^ contained three genes encoding cold shock proteins, *cspL*, *cspJ1*, and *cspJ2* (Table S1). These genes may help strain JC4^T^ grow in low temperatures. The heavy metal overload in aquatic ecosystems inhibits the growth of living organisms. Several bacteria are known to adapt to toxic heavy metals in polluted environments. Strain JC4^T^ has many genes and clusters related to the transport and resistance to the cations (As^3+^, Zn^2+^, Cu^2+^), such as cadmium, cobalt and zinc/H(+)-K(+) antiporter, arsenical-resistance protein, and cation efflux system protein (Table S2). Therefore, the strain may be able to tolerate multiple heavy metals and should be considered a candidate for bioremediation.

**Fig. 3:**
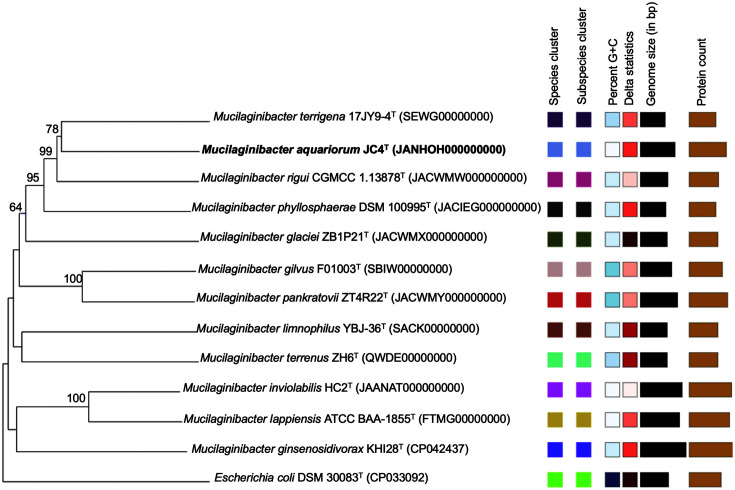
Tree inferred with FastME 2.1.6.1 from GBDP distances calculated from genome sequences. The branch lengths are scaled in terms of GBDP distance formula d5. The numbers above branches are GBDP pseudo-bootstrap support values > 60 % from 100 replications, with an average branch support of 73.2 %. The tree was rooted at the midpoint.

Strain JC4^T^ formed a cluster with *M. terrigena* 17JY9-4^T^ in the phylogenomic tree, confirming the classification of strain JC4^T^ to the genus *Mucilaginibacter* (Fig. 3). The ANI and dDDH values, when compared between strain JC4^T^ and its close relatives in the genus *Mucilaginibacter*, were below 80.92% and 23.9%, respectively (Table 1). These values were much lower than the thresholds for species delineation 95–96% for ANI and 70% for dDDH, revealing the genetic differences of strain JC4^T^ from other *Mucilaginibacter* species [52-54].

### Cell Morphology and Physiology

Strain JC4^T^ cells were Gram-negative and catalase/oxidase-positive, with a size of 0.3–0.5 μm in length and 0.1–0.2 μm in width (Fig. S3). The colonies on R2A medium were observed to be light pink-colored, smooth, circular, and convex with entire margins. The strain grew well on all tested media. Growth occurred at temperature of 4–30°C (optimum 20–30°C), at pH 5.0–8.5 (optimum 6.5–7.5), and at NaCl concentrations ranging within 0–1.0%(w/v) (optimum 0%). Strain JC4^T^ shared several similar morphological and physiological features with reference strains. For example, enzyme profiles of strain JC4^T^ exhibited a similar spectrum to its reference strains with the presence of alkaline phosphatase, valine arylamidase, and esterase (C4), etc. However, it could be distinguished from reference strains by the ability to reduce nitrates to nitrites, assimilate _L_-arabinose, hydrolyze skim milk, and produce acid from inulin (Table 2). The strain was sensitive to nalidixic acid, ciprofloxacin, tobramycin, norfloxacin, gentamicin, erythromycin, ampicillin, and amikacin. It exhibited resistance to cloxacillin, cefoperazone, penicillin, chloramphenicol, cefadroxil, vancomycin, ceftriaxone, amoxicillin, and ceftazidime. Details on the differential physiological and biochemical features of strain JC4^T^ compared with *M. rigui* KCTC 12534^T^, *M. lutimaris* KCTC 23461^T^, and *M. terrigena* KCTC 62294^T^ are shown in Table 2.

### Chemotaxonomic Characterization

Chemotaxonomic properties of strain JC4^T^ showed characteristic markers for the genus *Mucilaginibacter*. For instance, the primary fatty acids (> 10%) detected in strain JC4^T^ included summed feature 3 (C_16:1_ *ω7c* and/or C_16:1_ *ω6c*) (47.5%) and iso-C_15:0_ (16.8%) (Table 3). The presence of C_17:0_ cyclo distinguishes strain JC4^T^ from its closest phylogenetic neighbors. The polar lipid profile of JC4^T^ contained phosphatidylethanolamine, one unidentified phospholipid, one unidentified aminophospholipid, one unidentified aminolipid (AL3), and two unidentified lipids (L2, L4) as predominant polar lipids (Fig. S4). In addition, three unidentified aminolipids (AL1, AL2, and AL4) and four unidentified lipids (L1, L3, L5, and L6) in minor quantities were detected. Strain JC4^T^ is distinguished from the reference strains by the presence of an additional unidentified aminolipid as a major polar lipid. The respiratory quinone was MK-7.

## Conclusions

According to chemotaxonomic, phylogenetic, and phenotypic features, strain JC4^T^ belongs to the genus *Mucilaginibacter*. In addition, genome relatedness indices and several chemotaxonomic and phenotypic characteristics distinguish strain JC4^T^ from its closest phylogenetic neighbours. The polyphasic evidence presented here justifies the description of strain JC4^T^ as a new species in the genus *Mucilaginibacter*, and the name *Mucilaginibacter aquariorum* sp. nov. is proposed.

### Description of *M. aquariorum* sp. nov.

***M. aquariorum* (a.qua.ri.o’rum. N.L. gen. neut. pl. n. *aquariorum*, from/of aquaria).** Cells are Gram-negative, oxidase- and catalase-positive, non-motile, and rod-shaped. Colonies on R2A agar are pink in color, smooth, and round with entire margins. Capable of growth at pH 5.0–8.5 (optimum 6.5–7.5) and 4–37°C (optimum 20–30°C). The strain does not require NaCl for growth; however, it can tolerate NaCl concentrations up to 1.0%. Hydrolyze skim milk but not tween 20 and tween 80. In the API ZYM test, the following enzymatic reactions are positive: alkaline phosphatase, esterase (C4), esterase lipase (C8), leucine arylamidase, valine arylamidase, cystine arylamidase, acid phosphatase, naphthol-AS-BI-phosphohydrolase, α-galactosidase, β-galactosidase, α-glucosidase, β-glucosidase, N-acetyl-*β*-glucosaminidase, α-mannosidase, and α-fucosidase. In the API 20NE test, positive reactions are observed for reduction of nitrates to nitrites, hydrolysis of esculin, β-galactosidase, and assimilation of D-glucose, L-arabinose, D-mannose, and maltose. In API 50 CH tests, cells are positive for utilization of galactose, glucose, fructose, mannose, α-methyl-D-mannoside, α-methyl-D-glucoside, N-acetyl-glucosamine, amygdalin, arbutin, esculin, salicin, cellobiose, maltose, lactose, melibiose, sucrose, trehalose, inulin, raffinose, gentiobiose, and D-turanose as a sole carbon source. The major cellular fatty acids are summed feature 3 (C_16:1_ *ω7c* and/or C_16:1_ *ω6c*) and iso-C_15:0_. The major respiratory quinone is MK7. The predominant polar lipids consist of phosphatidylethanolamine, one unidentified phospholipid, one unidentified aminophospholipid, one unidentified aminolipid, and two unidentified lipids. The DNA G+C content of the type strain is 42.44%.

The type strain is JC4^T^ (= KCTC 92230^T^ = LMG 32715^T^), isolated from fresh water. The GenBank/EMBL/DDBJ accession numbers for the 16S rRNA gene and the draft genome sequence of type strain JC4^T^ are OP055897 and JANHOH000000000, respectively.

## Supplemental Materials

Supplementary data for this paper are available on-line only at http://jmb.or.kr.

## Figures and Tables

**Table 1:** Genome comparison of strain JC4^T^ with reference genomes of related taxa.

Species	Strain	Accession No.	Size (Mb)	G+C (%)	Gene	Protein	ANI (%)	dDDH (%)
*M. aquariorum*	JC4	JANHOH000000000	5.97	42.4	5,381	5,312	_	_
*M. rigui*	CGMCC 1.13878	JACWMW000000000	4.71	42.8	4,230	4,141	80.39	23.7
*M. terrigena*	17JY9-4	SEWG00000000	4.28	44.4	3,869	3,805	80.92	23.9
*M. phyllosphaerae*	DSM 100995	JACIEG000000000	4.42	43.7	3,937	3,872	79.35	22.5
*M. glaciei*	ZB1P21	JACWMX000000000	4.60	43.6	4,146	4,054	75.56	20.1
*M. gilvus*	F01003	SBIW00000000	5.42	44.8	4,809	4,723	75.32	20.1
*M. pankratovii*	ZT4R22	JACWMY000000000	6.37	45.3	5,518	5,409	75.25	20.0
*M. lappiensis*	ATCC BAA-1855	FTMG00000000	6.79	41.7	5,715	5,634	73.11	19.9
*M. limnophilus*	YBJ-36	SACK00000000	4.63	42.8	4,159	4,086	71.73	18.4
*M. terrenus*	ZH6	QWDE00000000	4.62	44.0	4,165	4,098	73.31	18.9
*M. ginsenosidivorax*	KHI28	CP042437	7.81	43.1	6,439	6,326	73.05	19.7
*M. inviolabilis*	HC2	JAANAT000000000	7.15	42.0	6,091	5,999	72.92	19.7

**Table 2:** Differential phenotypic characteristics of strain JC4^T^ and closely related strains.

Characteristics	1	2	3	4
Growth for				
Temperature range (°C)	4-30	4-37^†^	4-37^#^	4-30^††^
Optimum temperature (°C)	20–30	25^†^	257^#^	NA
pH range	5.0-8.5	5.0-10.0^†^	4.5-9.0^#^	6.0-8.0^††^
Optimum pH	6.5-7.5	6.0-7.0^†^	6.5-7.0^#^	NA
Hydrolysis of				
Skim milk	+	-	-	-
Tween 80	-	+	+	-
Biochemical characteristics (API ZYM, API 20E)				
Reduction of nitrates to nitrites	+	-	-	-
Assimilation of L-arabinose	+	-	-	-
Assimilation of *N*-acetyl-glucosamine	-	+	+	+
Esterase lipase (C8)	+	-	+	-
Production acid from (API 50 CH)				
Galactose	+	+	+	-
Fructose	+	-	-	-
α-Methyl-D-mannoside	+	+	+	-
α-Methyl-D-glucoside	+	+	+	-
Amygdalin	+	+	+	-
Arbutin	+	+	+	-
Salicin	+	+	+	-
Inulin	+	-	-	-
Main polar lipids	PE, 1PL, 1APL, 1AL, 2L	PE, 1APL^#^	PE, 1APL^#^	PE^††^

Strains: 1, JC4^T^; 2, *M. rigui* KCTC 12534^T^; 3, *M. lutimaris* KCTC 23461^T^; 4, *M. terrigena* KCTC 62294^T^. All data were obtained in this study, except where indicated otherwise. +, Positive; −, negative; NA, data not available. ^†^ data was obtained from [14]; ^#^ data was obtained from [5]; ^††^ data was obtained from [15]. PE, phosphatidylethanolamine; PL, unidentified phospholipid; APL, unidentified aminophospholipid; AL, unidentified aminolipid; L, unidentified lipid.

**Table 3:** Fatty acid compositions (%) of strain JC4^T^ and closely related type strains.

Fatty acids	1	2	3	4
iso-C_15:0_ 3-OH	3	2.1	1.9	2.2
C_15:0_ 2-OH	TR	1.1	TR	1.1
iso-C_15:0_	**16.8**	**18.6**	**21.1**	**23.4**
C_15:1_*ω6c*	TR	TR	TR	1.0
iso-C_16:0_	TR	1.3	1.2	1.1
C_16:1_*ω5c*	3.8	5.1	7.8	6.7
C_16:0_	3.4	3.2	3.8	TR
C_16:0_ 3-OH	TR	1.3	TR	TR
C_17:0_ cyclo	1.0	ND	ND	ND
C_17:1_*ω8c*	TR	1.2	TR	1.4
iso-C_17:0_ 3-OH	9.3	8.0	8.3	7.6
Summed Feature 3*	**47.5**	**47.7**	**40.7**	**44.4**
Summed Feature 4*	1.1	ND	1.4	1.0
Summed Feature 9*	6.5	4.9	5.0	5.3

Strains: 1, JC4^T^; 2, *M. rigui* KCTC 12534^T^; 3, *M. lutimaris* KCTC 23461^T^; 4, *M. terrigena* KCTC 62294^T^. Values were percentages of the total fatty acids. All data were obtained in this study, except where indicated otherwise. Fatty acids representing less than 1% of the total fatty acids in all strains were not listed. The proportions of the major fatty acids (>10.0%) are highlighted in bold. TR, trace amount (<1%); ND, not detected.

*Summed features are fatty acids that cannot be resolved reliably from another fatty acid using the chromatographic conditions chosen. The _MIDI_ system groups these fatty acids together as one feature with a single percentage of the total. Summed feature 3 contained C_16:1_ *ω6c*/C_16:1_ *ω7c*; summed feature 4 contained iso-C_17:1_ I/anteiso-C_17:1_ B; summed feature 9 contained 10-methyl C_16:0_ and/or iso-C_17:1_*ω9c*.

## References

[1] Pankratov TA, Tindall BJ, Liesack W, Dedysh SN (2007). *Mucilaginibacter paludis* gen. nov., sp. nov. and *Mucilaginibacter gracilis* sp. nov., pectin-, xylan- and laminarin-degrading members of the family *Sphingobacteriaceae* from acidic *Sphagnum* peat bog. Int. J. Syst. Evol. Microbiol..

[2] Lee SA, Le VV, Ko SR, Lee N, Oh HM, Ahn CY (2021). *Mucilaginibacter inviolabilis* sp. nov., isolated from the phycosphere of *Haematococcus lacustris* NIES 144 culture. Int. J. Syst. Evol. Microbiol..

[3] Kang H, Kim H, Bae S, Joh K (2021). *Mucilaginibacter aquatilis* sp. nov., *Mucilaginibacter arboris* sp. nov., and *Mucilaginibacter ginkgonis* sp. nov., novel bacteria isolated from freshwater and tree bark. Int. J. Syst. Evol. Microbiol..

[4] Yoon JH, Kang SJ, Park S, Oh TK (2012). *Mucilaginibacter litoreus* sp. nov., isolated from marine sand. Int. J. Syst. Evol. Microbiol..

[5] Kim JH, Kang SJ, Jung YT, Oh TK, Yoon JH (2012). *Mucilaginibacter lutimaris* sp. nov., isolated from a tidal flat sediment. Int. J. Syst. Evol. Microbiol..

[6] Choi L, Zhao X, Song Y, Wu M, Wang G, Li M (2020). *Mucilaginibacter hurinus* sp. nov., isolated from briquette warehouse soil. Arch. Microbiol..

[7] Li YP, You LX, Yang XJ, Yu YS, Zhang HT, Yang B (2022). Extrapolymeric substances (EPS) in *Mucilaginibacter rubeus* P2 displayed efficient metal(loid) bio-adsorption and production was induced by copper and zinc. Chemosphere.

[8] Fan X, Tang J, Nie L, Huang J, Wang G (2018). High-quality-draft genome sequence of the heavy metal resistant and exopolysaccharides producing bacterium *Mucilaginibacter pedocola* TBZ30T 06. Stand. Genomic Sci..

[9] Smith DL, Smith DL (2022). *Mucilaginibacter* sp. K improves growth and induces salt tolerance in nonhost plants via multilevel mechanisms. Front. Plant Sci..

[10] Wang ZY, Wang RX, Zhou JS, Cheng JF, Li YH (2020). An assessment of the genomics, comparative genomics and cellulose degradation potential of *Mucilaginibacter polytrichastri* strain RG4-7. Bioresour. Technol..

[11] Lin L, Yang H, Xu X (2022). Effects of water pollution on human health and disease heterogeneity: a review. Front. Environ. Sci..

[12] Yin H, Niu J, Ren Y, Cong J, Zhang X, Fan F (2015). An integrated insight into the response of sedimentary microbial communities to heavy metal contamination. Sci. Rep..

[13] Le VV, Ko SR, Kang M, Oh HM, Ahn CY (2022). *Hymenobacter cyanobacteriorum* sp. nov., isolated from a freshwater reservoir during the cyanobacterial bloom period. Arch. Microbiol..

[14] Baik KS, Park SC, Kim EM, Lim CH, Seong CN (2010). *Mucilaginibacter rigui* sp. nov., isolated from wetland freshwater, and emended description of the genus *Mucilaginibacter*. Int. J. Syst. Evol. Microbiol..

[15] Ten LN, Jeon NY, Li W, Cho YJ, Kim MK, Lee SY (2019). *Mucilaginibacter terrigena* sp. nov., a novel member of the family *Sphingobacteriaceae*. Curr. Microbiol..

[16] Ko SR, Le VV, Jin L, Lee SA, Ahn CY, Oh HM (2021). *Mariniflexile maritimum* sp. nov., isolated from seawater of the South Sea in the Republic of Korea. Int. J. Syst. Evol. Microbiol..

[17] Weisburg WG, Barns SM, Pelletier DA, Lane DJ (1991). 16S ribosomal DNA amplification for phylogenetic study. J. Bacteriol..

[18] Saitou N, Nei M (1987). The neighbor-joining method: a new method for reconstructing phylogenetic trees. Mol. Biol. Evol..

[19] Felsenstein J (1981). Evolutionary trees from DNA sequences: a maximum likelihood approach. J. Mol. Evol..

[20] Nei M, Kumar S, Takahashi K (1998). The optimization principle in phylogenetic analysis tends to give incorrect topologies when the number of nucleotides or amino acids used is small. Proc. Natl. Acad. Sci. USA.

[21] Kumar S, Stecher G, Li M, Knyaz C, Tamura K (2018). MEGA X: molecular evolutionary genetics analysis across computing platforms. Mol. Biol. Evol..

[22] Kimura M (1983). The Neutral Theory of Molecular Evolution.

[23] Le VV, Ko S-R, Lee S-A, Jin L, Blom J, Ahn C-Y (2021). *Cochlodiniinecator piscidefendens* gen. nov., sp. nov., an algicidal bacterium against the ichthyotoxic dinoflagellate *Cochlodinium polykrikoides*. Int. J. Syst. Evol. Microbiol..

[24] Bolger AM, Lohse M, Usadel B (2014). Trimmomatic: a flexible trimmer for Illumina sequence data. Bioinformatics.

[25] Simão FA, Waterhouse RM, Ioannidis P, Kriventseva E V, Zdobnov EM (2015). BUSCO: assessing genome assembly and annotation completeness with single-copy orthologs. Bioinformatics.

[26] Davis JJ, Wattam AR, Aziz RK, Brettin T, Butler R, Butler RM (2020). The PATRIC bioinformatics resource center: expanding data and analysis capabilities. Nucleic Acids Res..

[27] Zhao Y, Wu J, Yang J, Sun S, Xiao J, Yu J (2012). PGAP: pan-genomes analysis pipeline. Bioinformatics.

[28] Cantalapiedra CP, Herņandez-Plaza A, Letunic I, Bork P, Huerta-Cepas J (2021). eggNOG-mapper v2: functional annotation, orthology assignments, and domain prediction at the metagenomic scale. Mol. Biol. Evol..

[29] Blin K, Shaw S, Steinke K, Villebro R, Ziemert N, Lee SY (2019). AntiSMASH 5.0: updates to the secondary metabolite genome mining pipeline. Nucleic Acids Res..

[30] Yoon SH, Ha S min, Lim J, Kwon S, Chun J (2017). A large-scale evaluation of algorithms to calculate average nucleotide identity. Antonie Van Leeuwenhoek.

[31] Meier-Kolthoff JP, Auch AF, Klenk HP, Göker M (2013). Genome sequence-based species delimitation with confidence intervals and improved distance functions. BMC Bioinformatics.

[32] Meier-Kolthoff JP, Göker M (2019). TYGS is an automated high-throughput platform for state-of-the-art genome-based taxonomy. Nat. Commun..

[33] Smibert RM, Krieg NR, Gerhardt P, Murray RGE, Wood WA, Krieg NR (1994). Phenotypic characterization. Methods for General and Molecular Bacteriology.

[34] Bauer AW, Kirby WM, Sherris JC, Turck M (1966). Antibiotic susceptibility testing by a standardized single disk method. Am. J. Clin. Pathol..

[35] Sasser M (1990). Identification of Bacteria by Gas Chromatography of Cellular Fatty Acids, MIDI Technical Note 101.

[36] Minnikin DE, O'Donnell AG, Goodfellow M, Alderson G, Athalye M, Schaal A (1984). An integrated procedure for the extraction of bacterial isoprenoid quinones and polar lipids. J. Microbiol. Methods.

[37] Tindall BJ, Sikorski J, Smibert RA, Krieg NR, Reddy CA, Beveridge TJ, Breznak JA, Marzluf GA, Schmidt TM, Snyder LR Phenotypic characterization and the principles of comparative systematics. Methods for General and Molecular Bacteriology, 3rd Ed.

[38] Kates M (1972). Techniques of Lipidology: Isolation, Analysis and Identification of Lipids.

[39] Oren A, Duker S, Ritter S (1996). The polar lipid composition of Walsby's square bacterium. FEMS Microbiol. Lett..

[40] Tamaoka J (1986). Analysis of bacterial menaquinone mixtures by reverse-phase high-performance liquid chromatography. Methods Enzymol..

[41] Chun J, Oren A, Ventosa A, Christensen H, Arahal DR, da Costa MS (2018). Proposed minimal standards for the use of genome data for the taxonomy of prokaryotes. Int. J. Syst. Evol. Microbiol..

[42] Osbourn A (2010). Secondary metabolic gene clusters: evolutionary toolkits for chemical innovation. Trends Genet..

[43] Barbosa J, Caetano T, Mendo S (2015). Class I and class II lanthipeptides produced by Bacillus spp. J. Nat. Prod..

[44] Oldfield E, Lin FY (2012). Terpene biosynthesis: modularity rules. Angew. Chemie - Int. Ed..

[45] Ay H (2020). *Nonomuraea terrae* sp. nov., isolated from arid soil. Arch. Microbiol..

[46] Le VV, Ko SR, Lee SA, Kang M, Oh HM, Ahn CY (2022). *Caenimonas aquaedulcis* sp. nov., isolated from freshwater of Daechung Reservoir during *Microcystis* bloom. J. Microbiol. Biotechnol..

[47] Le VV, Ko SR, Kang M, Lee SA, Oh HM, Ahn CY (2022). *Panacibacter microcysteis* sp. nov., isolated from a eutrophic reservoir during the *Microcystis* bloom period. Arch. Microbiol..

[48] Nouioui I, Pötter G, Jando M, Goodfellow M (2022). *Nocardia noduli* sp. nov., a novel actinobacterium with biotechnological potential. Arch. Microbiol..

[49] Yamada Y, Kuzuyama T, Komatsu M, Shin-ya K, Omura S, Cane DE (2015). Terpene synthases are widely distributed in bacteria. Proc. Natl. Acad. Sci. USA.

[50] Agrawal S, Acharya D, Adholeya A, Barrow CJ, Deshmukh SK (2017). Nonribosomal peptides from marine microbes and their antimicrobial and anticancer potential. Front. Pharmacol..

[51] Keto-Timonen R, Hietala N, Palonen E, Hakakorpi A, Lindström M, Korkeala H (2016). Cold shock proteins: a minireview with special emphasis on Csp-family of enteropathogenic Yersinia. Front. Microbiol..

[52] Kim M, Oh HS, Park SC, Chun J (2014). Towards a taxonomic coherence between average nucleotide identity and 16S rRNA gene sequence similarity for species demarcation of prokaryotes. Int. J. Syst. Evol. Microbiol..

[53] Auch AF, von Jan M, Klenk HP, Göker M (2010). Digital DNA-DNA hybridization for microbial species delineation by means of genome-to-genome sequence comparison. Stand. Genomic Sci..

[54] Richter M, Rossell-Móra R (2009). Shifting the genomic gold standard for the prokaryotic species definition. Proc. Natl. Acad. Sci. USA.

