# Supplementary material for: Mucilaginibacter aquariorum sp. nov., Isolated from Fresh Water
Source: J Microbiol Biotechnol. 2022 Oct 31;32(12):1553–60. doi: 10.4014/jmb.2208.08021 (PMC9843747; doi:10.4014/jmb.2208.08021)
Supplement: Supplementary file 1 [file jmb-32-12-1553-supple.pdf]

J. Microbiol. Biotechnol.
https://doi.org/10.4014/jmb.2208.08021

Supplementary Materials

Mucilaginibacter aquariorum sp. nov., isolated from fresh water

Ve Van Le[1,2], So-Ra Ko[1], Mingyeong Kang[1,2], Hee-Mock Oh[1,2], and Chi-Yong Ahn[1,2*]

[1]Cell factory Research Centre, Korea Research Institute of Bioscience & Biotechnology, 125 Gwahak-ro, Yuseong-gu, Daejeon 34141, Republic of Korea

[2]Department of Environmental Biotechnology, KRIBB School of Biotechnology, University of Science and Technology, Daejeon 34113, Republic of Korea

[*]Corresponding authors

Chi-Yong Ahn

Tel: +82-42-860-4329

Fax: +82-42-860-4594

E-mail: cyahn@kribb.re.kr

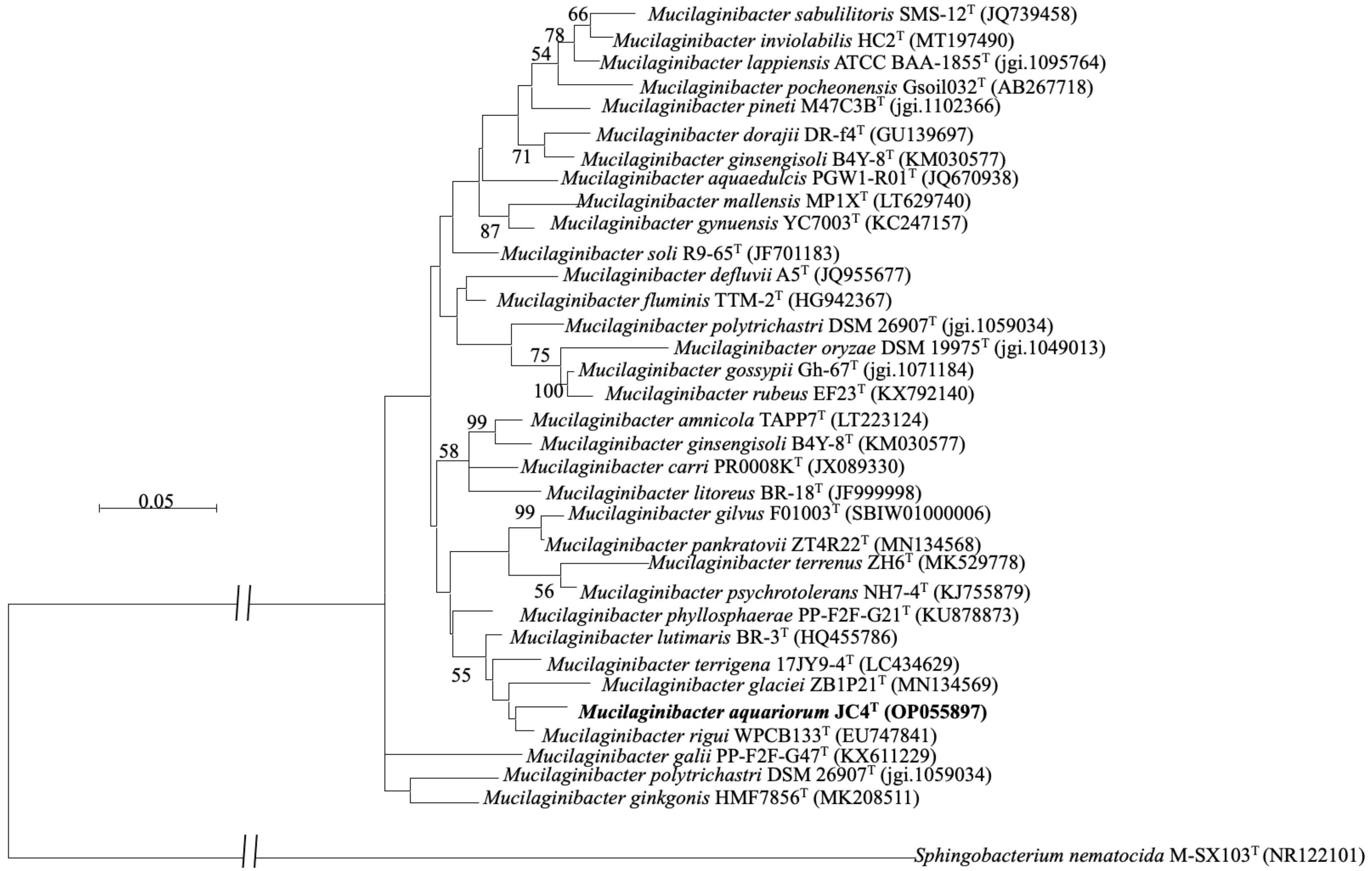

Fig. S1. Maximum-likelihood phylogenetic tree based on the 16S rRNA gene sequences depicting the position of strain JC4^{T} among the

related members of the genus *Mucilaginibacter*. Bootstrap values (≥ 50%) based on 1,000 replications are shown at branch points.

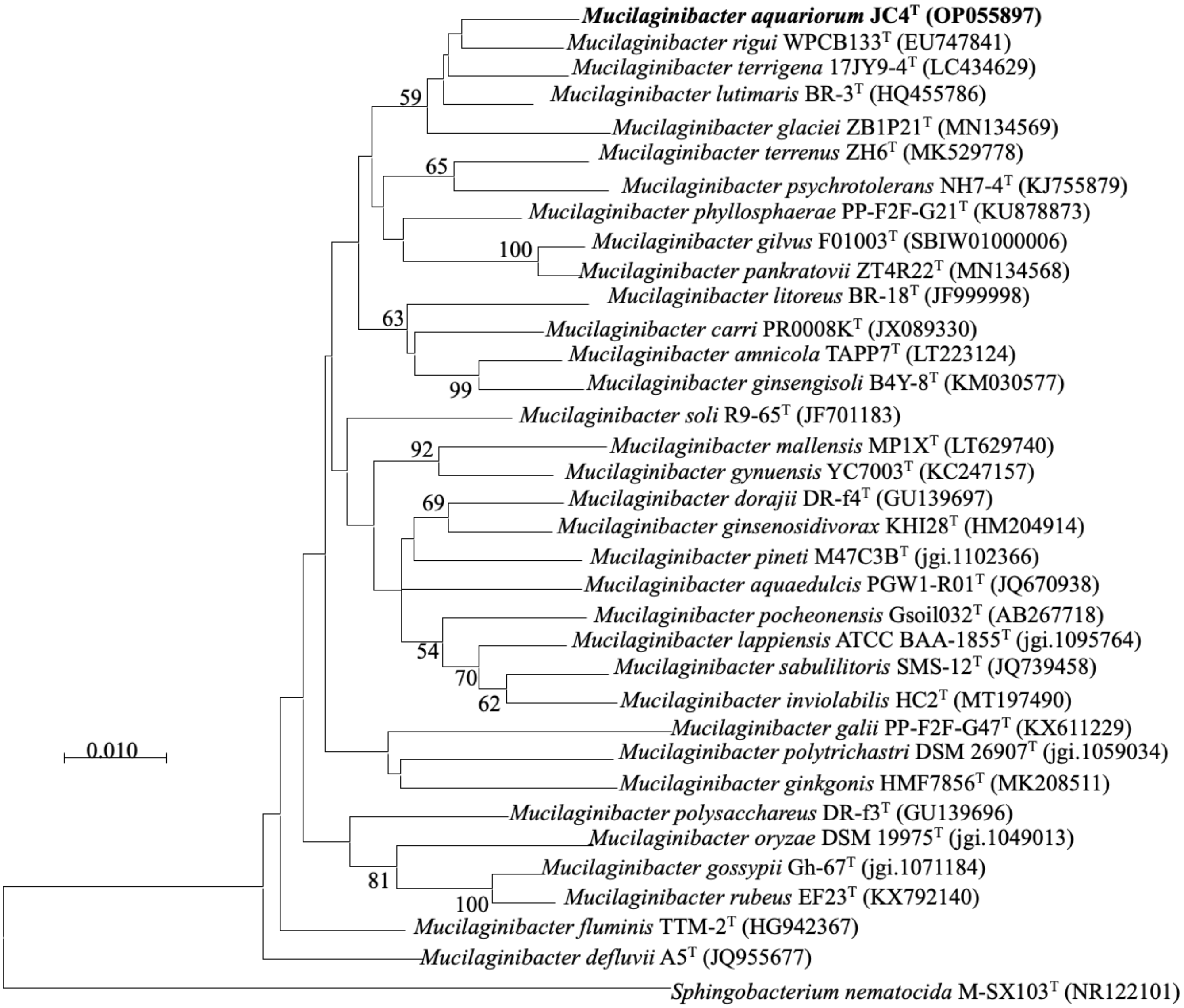

Fig. S2. Minimum evolution phylogenetic tree based on the 16S rRNA gene sequences depicting the position of strain JC4^{T} among the related members of the genus *Mucilaginibacter*. Bootstrap values (≥ 50%) based on 1,000 replications are shown at branch points.

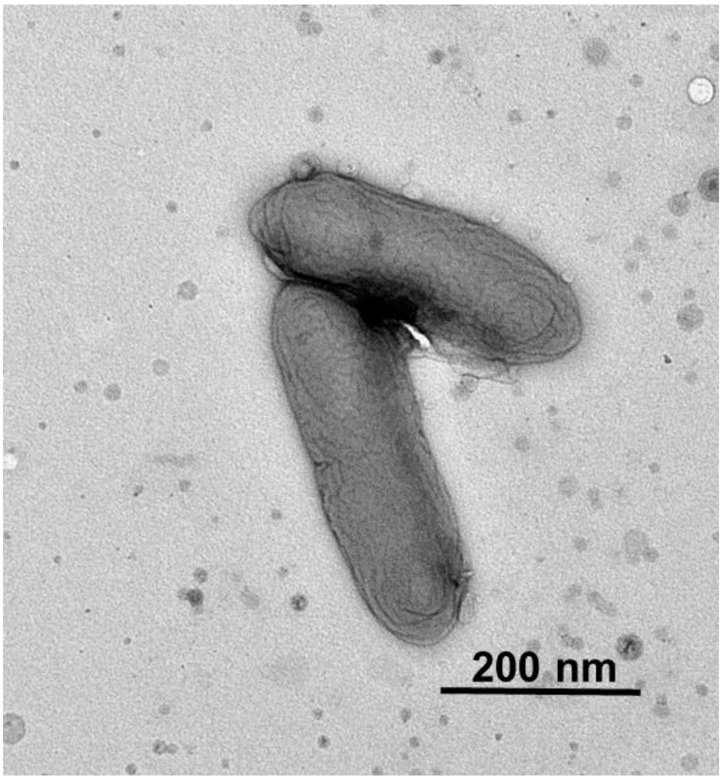

Fig. S3. Morphology of strain JC4^T. Transmission electron micrograph: bar, 200 nm. The cells were grown on R2A at 25℃ for 3 days.

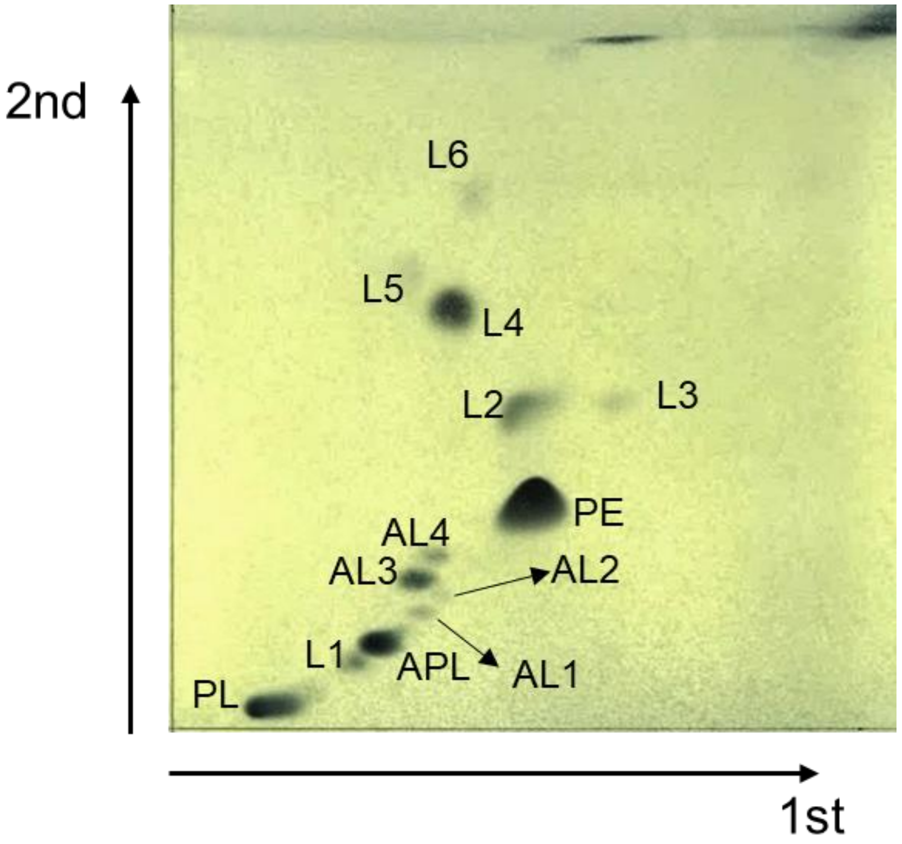

Fig. S4. Two-dimensional thin layer chromatography of polar lipids extracted from strain JC4[T]. PE, phosphatidylethanolamine; PL, unidentified phospholipid; APL, unidentified aminophospholipid; AL, unidentified aminolipid; L, unidentified lipid.

Table S1. Putative genes involved in cold tolerance

**Locus_Tag**	**Start**	**End**	**Strand**	**Product**	**Gene**
JC4_00534	618786	618983	+	Cold shock protein 2	*cspL*
JC4_02811	617370	617561	-	Cold shock-like protein CspJ	*cspJ1*
JC4_03036	873930	874124	+	Cold shock-like protein CspJ	*cspJ2*

Table S2. Putative genes involved in heavy metals resistance

Locus_Tag	Start	End	Strand	Product	Gene
JC4_04846	62124	63401	+	Cation efflux system protein CusA	*cusA1*
JC4_04847	63398	65308	+	Cation efflux system protein CusA	*cusA2*
JC4_01930	2191007	2191921	+	Cadmium, cobalt and zinc/H(+)-K(+) antiporter	*czcD*
JC4_02804	607698	608969	+	Cobalt-zinc-cadmium resistance protein CzcC	*czcC*
JC4_02805	608997	610091	+	Cobalt-zinc-cadmium resistance protein CzcB	*czcB*
JC4_02806	610151	613288	+	Cobalt-zinc-cadmium resistance protein CzcA	*czcA1*
JC4_03052	5112	8165	+	Cobalt-zinc-cadmium resistance protein CzcA	*czcA2*
JC4_03228	178467	180356	+	Divalent metal cation transporter MntH	*mntH*
JC4_00396	475597	476523	+	putative metallo-hydrolase YflN	*yflN*
JC4_01186	1352070	1352903	-	Putative metallo-hydrolase YycJ	*yycJ*
JC4_01239	1423394	1425364	-	ATP-dependent zinc metalloprotease FtsH	*ftsH1*
JC4_02467	275229	277196	-	ATP-dependent zinc metalloprotease FtsH	*ftsH2*
JC4_02720	519040	519765	-	Metallo-beta-lactamase type 2	*blaB1*
JC4_02905	731884	733866	+	ATP-dependent zinc metalloprotease FtsH	*ftsH3*
JC4_03228	178467	180356	+	Divalent metal cation transporter MntH	*mntH*
JC4_03273	225954	228422	+	Metal-pseudopaline receptor CntO	*cntO1*
JC4_03947	114370	116004	-	Metalloprotease TldD	*tldD*
JC4_04798	16350	16877	+	Putative metal-dependent hydrolase YfiT	*yfiT*
JC4_04809	27371	29866	-	Metal-pseudopaline receptor CntO	*cntO2*
JC4_05170	103917	106028	+	ATP-dependent zinc metalloprotease FtsH	*ftsH4*
JC4_01108	1253065	1253826	+	Copper homeostasis protein CutC	*cutC*
JC4_01832	2079278	2079982	-	Fatty acyl-CoA reductase	*acr1*
JC4_02787	589548	590606	+	Arsenical-resistance protein Acr3	*acr3*
JC4_03285	244401	245630	+	Multidrug efflux pump subunit AcrA	*acrA*
JC4_02786	589073	589546	+	Arsenate reductase	*arsC*
JC4_02787	589548	590606	+	Arsenical-resistance protein Acr3	*acr3*
JC4_02880	705177	706943	-	Arsenical pump-driving ATPase	*arsA*
JC4_00067	77744	79876	-	putative copper-importing P-type ATPase A	*copA1*
JC4_01108	1253065	1253826	+	Copper homeostasis protein CutC	*cutC*
JC4_02966	804925	807315	+	Copper-exporting P-type ATPase	*copA2*
JC4_03004	841633	843687	-	Copper-exporting P-type ATPase B	*copB*
JC4_01930	2191007	2191921	+	Cadmium, cobalt and zinc/H(+)-K(+) antiporter	*czcD*
JC4_02804	607698	608969	+	Cobalt-zinc-cadmium resistance protein CzcC	*czcC*
JC4_02805	608997	610091	+	Cobalt-zinc-cadmium resistance protein CzcB	*czcB*
JC4_02806	610151	613288	+	Cobalt-zinc-cadmium resistance protein CzcA	*czcA1*
JC4_03052	5112	8165	+	Cobalt-zinc-cadmium resistance protein CzcA	*czcA2*
